# A Review of Recent Advances in Flexible Wearable Sensors for Wound Detection Based on Optical and Electrical Sensing

**DOI:** 10.3390/bios12010010

**Published:** 2021-12-23

**Authors:** Xianyou Sun, Yanchi Zhang, Chiyu Ma, Qunchen Yuan, Xinyi Wang, Hao Wan, Ping Wang

**Affiliations:** 1 Biosensor National Special Laboratory, Key Laboratory for Biomedical Engineering of Ministry of Education, Department of Biomedical Engineering, Zhejiang University, Hangzhou 310027, China; 12015014@zju.edu.cn (X.S.); 22115053@zju.edu.cn (Y.Z.); mcy1996@zju.edu.cn (C.M.); sumyuan@zju.edu.cn (Q.Y.); wxy317@zju.edu.cn (X.W.); 2 Binjiang Institute of Zhejiang University, Hangzhou 310053, China

**Keywords:** flexible wearable sensors, smart bandages, biosensors, wound detection

## Abstract

Chronic wounds that are difficult to heal can cause persistent physical pain and significant medical costs for millions of patients each year. However, traditional wound care methods based on passive bandages cannot accurately assess the wound and may cause secondary damage during frequent replacement. With advances in materials science and smart sensing technology, flexible wearable sensors for wound condition assessment have been developed that can accurately detect physiological markers in wounds and provide the necessary information for treatment decisions. The sensors can implement the sensing of biochemical markers and physical parameters that can reflect the infection and healing process of the wound, as well as transmit vital physiological information to the mobile device through optical or electrical signals. Most reviews focused on the applicability of flexible composites in the wound environment or drug delivery devices. This paper summarizes typical biochemical markers and physical parameters in wounds and their physiological significance, reviews recent advances in flexible wearable sensors for wound detection based on optical and electrical sensing principles in the last 5 years, and discusses the challenges faced and future development. This paper provides a comprehensive overview for researchers in the development of flexible wearable sensors for wound detection.

## 1. Introduction

A wound is a common pathological condition of the skin, which has a wide range of sources, including accidental skin damage, related diseases (such as diabetes, cancer, and vascular disease), and conventional medical surgery [1]. According to the source and the healing rate, wounds can be divided into acute and chronic wounds [2]. Although mild wounds are usually easy to recover for healthy people, the healing processes of larger or deeper skin injuries and the chronic wounds caused by the disease tend to be problematic [3]. On the one hand, they cause physical pain to patients, reducing their quality of life and even putting them at risk of amputation or death [4]. For example, diabetic foot ulcer (DFU) is very difficult to heal, and more than 12% of DFUs may lead to lower-limb amputations. Moreover, the International Diabetes Foundation estimates that the global prevalence of diabetes will reach 9.9% by 2030, while people with diabetes have a 25% chance of developing DFUs in their lifetime [5]. It is no exaggeration to say that the number of patients with chronic wounds is going to surge without rational control. Chronic wounds, on the other hand, take huge medical costs, with an estimated 1.5–2 million people suffering from chronic wounds across Europe; in the United States, where chronic wounds affect 6.5 million lives, the cost of treating chronic wounds exceeds 25 billion USD a year [6]. However, statistically consumptive wound dressing is not the major component among the vast expenditure; nursing and hospitalization together account for 80–85% of the total cost of wound management because of the cumbersome care for the complex process of chronic wound healing [7].

In general, the healing process of a wound is divided into four stages: hemostasis, inflammation, proliferation, and maturation [8,9]. The hemostasis stage is the body′s immediate response to injury, where platelets help form a hemostatic plug to reduce bleeding [10]. During the inflammation stage, vascular permeability increases, allowing enzymes, nutrients, and immune cells to reach the site of injury. Circulating inflammatory cells remove damaged cells and microorganisms from the wound site [11]. During the proliferation stage, granulation tissue is formed and the integrity of the skin is restored, allowing it to act as a barrier again [12]. Tissue maturation is the stage of transformation of granulation tissue into scar, degeneration of vascular network, and extensive replacement and remodeling of the extracellular matrix and collagen [11]. If each stage is completed properly, then the wound will heal successfully (acute wound). However, if at least one of these stages cannot be completed properly due to some reason such as repeated bacterial infection or physical illness, the wound will persist for a long time, resulting in a chronic wound [13,14,15,16]. Unfortunately, chronic wounds are often prone to bacterial infections due to protein-rich and oxygen-deprived environments [17], creating a vicious cycle. For the treatment of chronic wounds, the traditional program has many disadvantages. Firstly, the current qualitative assessment of wounds through visual examination is subjective and susceptible to personal experience [18], and some wounds (e.g., pressure sores) are invisible to the naked eye at an early stage of formation [19]. Furthermore, people usually cover the wound with a suitable wound dressing for interventional treatment of the wound [20,21]. However, due to frequent changes in wound healing and the limited medication contained, the wound dressing is required to be replaced promptly. Frequent dressing changes not only cause pain to patients but also have a severe effect on wound healing and even secondary trauma [22,23]. Therefore, it is imperative to find a new high-efficiency, low-cost method for wound detection and recovery through technological innovation.

**Figure 1:**
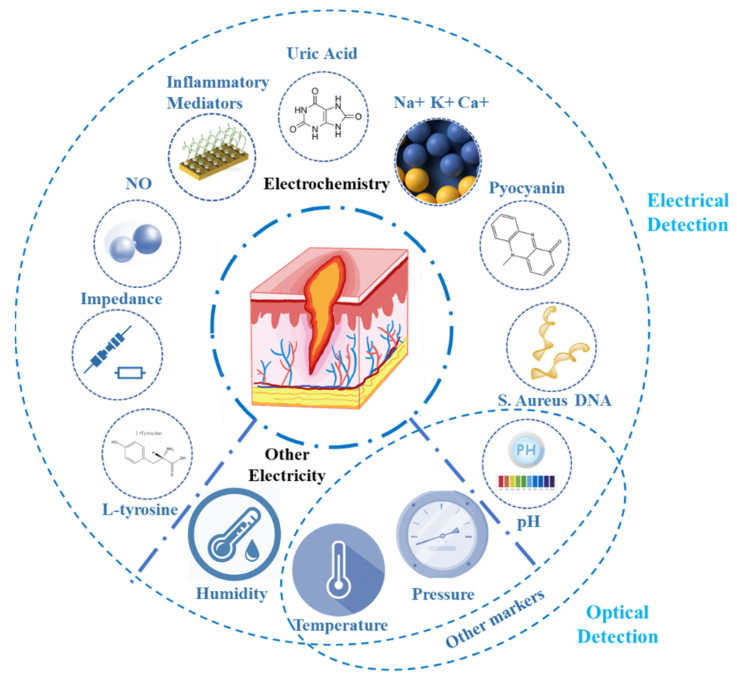
Key markers of wounds detected by flexible wearable sensors based on electrical and optical principles involved in this paper. Among them, electrochemical sensors are widely used for quantitatively detecting a variety of markers.

Fortunately, with the emergence of flexible electrodes and the advancement of smart sensing technology, the concept of wearable smart bandages has been proposed in recent years [24]. Sensors or drug release devices are integrated into a bandage dressing with good biocompatibility for wound care. Smart bandages can detect useful biochemical or physical information in the wound without disassembling the bandage and transmit to external devices, thus avoiding unnecessary damage caused by frequently opening the bandage to check the wound status. The use of smart bandages for early identification of bacterial infections can reduce the probability of chronic wounds and improve wound healing efficiency. Some recent studies implemented bandages to autonomously release drugs according to the real-time physiological conditions of the wound [25], forming a complete closed-loop of wound detection and treatment, which would be highly beneficial for patients suffering from chronic wounds. Although other review articles also mentioned smart bandages [26,27,28,29,30,31,32], they mainly focused on the applicability of flexible composites in the wound environment or drug delivery devices. Considering that the detection function is the foundation of the entire smart bandage system, we provide a comprehensive and detailed summary of the key markers in wounds and the methods of wound detection by flexible wearable sensors (Figure 1) based on optical and electrical sensing. Since electrical-based methods allow for precise quantitative measurements compared to optical-based methods, which are necessary for real clinical applications, we focus on reviewing electrical detection methods, especially electrochemical detection, and we discuss the current challenges and future directions of these efforts.

The abbreviations covered in this paper are summarized in Abbreviation table.

## 2. Key Markers of Wounds and Their Physiological Significance

It is an urgent task to warn of the emergence of chronic wounds. As the wound environment deteriorates and progressively develops into a chronic wound, certain markers in the wound environment will become abnormal. Commonly, the level or concentration of certain markers will change; for example, an increase in temperature or a sudden rise in pH is often indicative of a bacterial infection, which will increase the chance of chronic wound appearance [33,34]. However, the microenvironment in the wound is very complicated. Although many markers are related to the process of wound healing, most of them are only present in trace amounts in the wound, which are difficult to detect [35,36,37]. In this section, we summarize important wound markers including biochemical markers and physical markers, as well as their physiological significance in wounds.

### 2.1. The Physiological Significance of Biochemical Markers in Wounds

#### 2.1.1. The Physiological Significance of Uric Acid in Wounds

Uric acid is widely present in various human body fluids including the wound exudate [38]. Uric acid in wound exudate is related to oxidative stress and bacterial infection [39,40,41]. Some studies have confirmed that a higher concentration of uric acid in wound exudate denotes greater severity of the wound [42,43]. Generally, the concentration of uric acid in wound exudate varies between 220 and 750 mM/L, depending on the severity of the wound [44]. The cell damage caused by the wound will release adenosine triphosphate (ATP) into the extracellular matrix. ATP is then metabolized by various related enzymes, resulting in the production of uric acid [45]. A more serious wound features larger-scale cell damage and more ATP released to the extracellular matrix, which may lead to more uric acid production. In addition, other studies have proven that infection with *Staphylococcus aureus* (*S. aureus*) and *Pseudomonas aeruginosa* (*P. aeruginosa*) can also lead to a change in uric acid concentration in the wound [46,47,48,49]. In the process of bacterial infection, due to the catalysis of microbial uricase (UOx), uric acid will be decomposed into allantoin [50], resulting in a decrease in uric acid concentration [51,52]. Therefore, the absolute value of uric acid concentration and the fluctuation of uric acid concentration can be used to reflect the severity of the wounds and bacterial infection.

#### 2.1.2. The Physiological Significance of pH in Wounds

pH is one of the most important and commonly used parameters in wound condition assessment, and it plays a vital role in a variety of physiological processes in wounds. The pH of normal wounds is weakly acidic (about 5.5 to 6.5), which helps promote angiogenesis and epithelialization [53,54,55,56]. However, when bacteria infect a wound, the alkaline byproduct of bacterial proliferation will increase the pH of the wound, which can often exceed 7.3 in the presence of high bacterial levels [33]. When this happens, there are many physiological changes in the wound that are not conducive to healing, which is reflected in two aspects [57]. On the one hand, the alkaline wound environment can cause abnormalities in the enzymes that promote healing in the human body, such as matrix metalloproteinases and metalloproteinases. The presence of metalloproteinases can limit the overexpression of matrix metalloproteinases; hence, the balance between the two is essential for wound healing. However, the alkaline environment in the wound will break this balance, which may promote the overexpression of matrix metalloproteinases and make the wound difficult to heal [58,59]. On the other hand, an alkaline wound environment provides a good condition for bacterial proliferation and the expression of toxicity of bacterial secretions [60]. Therefore, pH can be used to evaluate the process of wound healing and indicate whether drugs are needed.

#### 2.1.3. The Physiological Significance of Other Biochemical Markers in Wounds

The two most common bacterial species in wound infections are *S. aureus* and *P. aeruginosa* [61,62,63,64]. Although uric acid can reflect the infection of bacteria in the wound, it cannot identify the specific bacteria, which may affect the accuracy of wound treatment decision making. Bacteria usually secrete a variety of biochemical byproducts to complete their physiological activities, and pyocyanin is a phenazine derivative with redox activity secreted by *P. aeruginosa* [65,66,67,68,69]. Due to its unique redox properties, pyocyanin easily reacts with the cell metabolism to generate reactive oxygen species, which will cause damage to host tissues [65,70,71]. Pyocyanin is a specific marker of *P. aeruginosa* [72]; thus, it can be used to reflect the severity of *P. aeruginosa* infection in wounds. It is reported that the biomedical-related concentration range of pyocyanin is 1–100 μM [61]. As for *S. aureus*, it has been reported that infection in wounds can be reflected by detecting its DNA molecules [73].

l-Tyrosine is a nonessential amino acid in the human body. However, related studies have shown that the concentration of l-tyrosine in the wound exudate of patients suffering from DFU will increase sharply [74], which indicates the deterioration of the condition. Therefore, as one of the few DFU wound markers, l-tyrosine has important physiological value in the evaluation of DFU wounds.

Nitric oxide (NO) is another biochemical marker in chronic wounds, which promotes the healing process of wounds [75,76]. NO is secreted by the host′s immune cells to resist infection by pathogens at the wounds [77,78]. In the presence of oxygen, NO will be converted into reactive nitrogen, which will cause damage to the biological macromolecules (such as proteins, DNA, and lipids) that invade pathogens to achieve the purpose of fighting infection [79]. Therefore, the detection of NO in chronic wounds can reflect the infection of pathogenic bacteria in the wounds to a certain extent. Some studies have shown that NO in chronic wounds can induce the dispersion of infectious *P. aeruginosa* cell membranes [80,81]. Although the study results of Simoska et al. [79] showed that there is no direct evidence that NO is resistant to *P. aeruginosa*, this is still an issue we should strive to explore.

In addition, Na^+^, K^+^, and Ca^2+^ can reflect the physiological state of the wounds [82]. Some related inflammatory mediators, including tumor necrosis factor-α, interleukin-6, interleukin-8, and transforming growth factor-β1, have also been reported for wound evaluation [83]. More biochemical markers deserve further exploration, as they represent the cornerstone for promoting the process of wound detection.

### 2.2. The Physiological Significance of Physical Parameters in Wounds

Some physical parameters in the wound can also directly or indirectly reflect the wound infection or healing. The more common ones are impedance, temperature, pressure, and humidity.

The impedance of skin and wound can be used for early pressure ulcer identification and evaluation of the wound healing process. Pressure ulcers are common chronic wounds, which occur when pressure is applied to a certain area of the body for a long time. Pressure ulcers usually occur where the bones of patients (such as patients with high paraplegia) who are unable to move autonomously are protruding. The tissues are squeezed by contact objects and bones, causing loss of blood flow and eventually tissue necrosis [84,85,86,87]. The early stages of pressure ulcers are often invisible to the naked eye, but the physiological state of the subcutaneous tissues can be reflected by skin impedance [88]. Therefore, skin impedance can be used to monitor the formation of early pressure ulcers so that countermeasures can be taken in advance. In addition, the impedance can also be used to monitor the change of wound area and the degree of healing, since impedance differs between wounds and normal skin, as well as for the same wound at different stages of healing [55].

Temperature is often used to reflect the infection of bacteria in the wound. When the temperature of the wound suddenly rises, it is considered that there is a high probability of bacterial infection. Usually, the temperature change threshold corresponding to the deterioration of the infection is about 2 °C [10,34,89]. The decrease in temperature at the wound may indicate local ischemia [90,91] and may also lead to a decrease in the activity of some enzymes, which affects the normal physiological activities of the wound and, thus, leads to difficulty in wound healing.

Humidity is also essential for wound healing, and both high and low humidity can affect wound healing. As most biochemical reactions involved in wounds are dependent on liquid media, adequate levels of humidity need to be maintained in the wound [31]. However, there are often high concentrations of protein-degrading enzymes, neutrophils, and proinflammatory cytokines in wound exudate; thus, too much wound exudate may cause the expansion of the wound instead of promoting wound healing [92,93]. Therefore, wound humidity monitoring is of great significance for the assessment of the wound microenvironment.

For some special wounds, such as venous ulcers, the pressure of the bandage on the skin and the wound needs to be precisely controlled [94,95], which is very important for the healing of such wounds. In addition, a pressure change at the wound can also reflect the degree of swelling of the wound.

It is worth mentioning that, for sophisticated detection systems [25], these physical parameters such as temperature and humidity can be used as sensor compensation to improve the accuracy of the sensor.

## 3. Key Wound Marker Detection

### 3.1. Electrical Detection of Wound Markers

Electrical detection methods can transform markers in wounds into precise, quantitative electrical signals, enabling the trace detection of markers in complex wound environments with high specificity and sensitivity [96]; they have attracted a great deal of interest from researchers. Moreover, the electrical signal can easily interact with other external devices such as smartphones, making information processing more efficient and intelligent.

#### 3.1.1. Electrochemical Detection

Electrochemistry is one of the most important methods of electrical detection, which uses chemical reactions to convert physiological information into precise electrical signals according to the properties of biochemical markers [51]. In addition, electrochemical impedance spectroscopy (EIS) supports the measurement of skin and wound impedance. Therefore, electrochemical methods are widely used in wound detection.

##### The Detection of Uric Acid

The real wound environment is very complicated. For the electrochemical detection of wound exudate, on the one hand, it is necessary to consider the impact of other oxidizing or reducing biomolecules on the target detection, such as ascorbic acid, tyrosine, or tryptophan [97]; on the other hand, it should be noted that some biological macromolecules may contaminate the electrodes and reduce the repeatability of the sensor. In addition, as a wearable sensor, the mechanical properties and wearing comfort of the bandage should also be considered while ensuring the sensing performance.

Since uric acid is inherently redox, specific oxidation peaks can be generated in electrochemical voltammetry and, thus, be measured. As early as 2008, Sharp et al. [97] explored a uric acid electrochemical sensor for wound detection. They constructed an electrochemical sensor with a carbon fiber mesh as the substructure via a laser etching process and used square wave voltammetry to assess the content of uric acid in wound exudates. The electrode was surface anodized in 0.1 M sodium hydroxide solution, which helped to increase the transfer rate of electrons, to better distinguish uric acid from other substances. Subsequently, cellulose acetate, which is a protective barrier for permeation, was modified on the electrode, repelling other biological macromolecules through the size effect to prevent them from contaminating the electrode, thereby improving the repeatability of the electrode. The fitted linear equation obtained by the sensor in the range of uric acid concentration of 0–500 μM was *y* = 0.025*x* + 0.561, R^2^ = 0.97. In the team′s follow-up study [98,99,100], the carbon polyethylene mesh electrodes were further developed and equipped with a miniature potentiostat to facilitate patient wear and use. It could generate 7174 μA current response for 1 mol/dm^3^ of uric acid.

**Figure 2:**
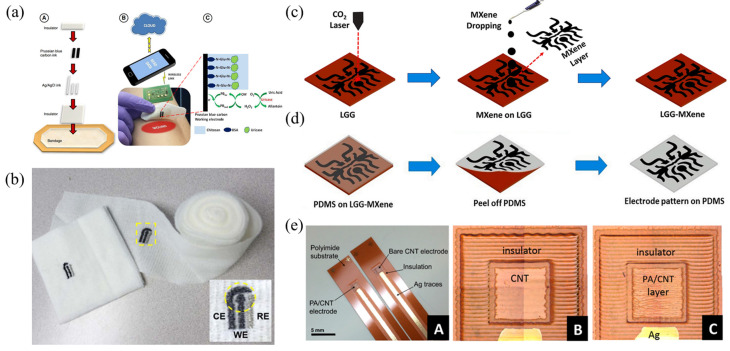
Wearable sensors for detecting uric acid levels in wounds. (**a**) Wearable sensor system based on UOx [62]. Copyright (2015) Elsevier. (**b**) Wearable uric acid detection sensor made by embroidery process on gauze [105]. Copyright (2017) Elsevier. (**c**) LGG-MXene electrode fabrication [106]. Copyright (2020) Elsevier. (**d**) Transferring the obtained LGG-MXene hybrid scaffold onto PDMS [106]. Copyright (2020) Elsevier. (**e**) Optical micrograph of two inkjet-printed electrodes on Kapton strips and laser scanning micrographs of the CNT and PA/CNT sensing areas [72]. Copyright (2019) American Chemical Society.

Although the electrode can distinguish the current peaks produced by uric acid after surface anodization, sensors based on this principle often require a large positive potential on the working electrode to catalyze the oxidation of uric acid, which may lead to the interference of other easily oxidized biomolecules (such as tyrosine and tryptophan) in the wound exudate, resulting in detection error [101]. To solve this problem, an electrochemical sensor based on UOx has been developed, which can provide better specificity and sensitivity. The UOx catalyzes the oxidation of uric acid to produce allantoin and hydrogen peroxide, which is more readily reduced, and the reduction current is recorded by an electrode for calculating the concentration of uric acid. Many methods and materials are available for catalytic reduction of hydrogen peroxide; thus, there is a wide scope for innovation for researchers. Kassal et al. [62] reported a new flexible wearable uric acid biosensor, which was made by screen-printing a carbon electrode modified with Prussian blue (PB) onto a commercial bandage, and immobilizing the UOx on the working electrode (Figure 2a). The enzyme can highly specifically oxidize uric acid to generate hydrogen peroxide, which was further catalyzed and reduced by the PB-carbon electrode. This allowed the sensor to detect uric acid very sensitively and specifically at a very low negative working potential (Ewrk = −0.3 V). Its sensitivity could reach −2.4 nA/μM uric acid, and it still had good selectivity to uric acid under the influence of other biomolecules (such as ascorbic acid).

Kassal’s study undoubtedly provides a reference for similar studies on the detection of uric acid in wounds. However, in this work, the method of immobilization of UOx on the electrode was not thoroughly explored; hence, the effect of uric acid oxidase may not be optimal, which affects the stability and repeatability of the sensor. In fact, there are many ways to modify enzymes on electrochemical electrodes, including physisorption, chemisorption, and entrapment [102]. RoyChoudhury et al. [103] reported a flexible wearable electrochemical sensor for uric acid detection based on UOx, and the UOx was entrapped in poly(vinyl alcohol) *N*-methyl-4(4′-formylstyryl) pyridinium methosulfate acetal, a cationic polymer matrix. In addition, ferrocene carboxylic acid (FCA) was also introduced to assist the electron transfer between the active site of the enzyme and the surface of the sensor. Various characterization methods pointed to the fact that the enzyme embedded in this cationic polymer was more stable and more uniform, thus improving the repeatability and stability of the sensor. Furthermore, the introduction of FCA could improve electron transport between the active site of the enzyme and the electrode, providing an enhanced response. The entrapped UOx biosensor could remain stable within 48 h and still maintained 90% activity until the fifth day. In the actual wound fluid and sweat spike recovery experiment, the recovery rate could reach 102–107%. In terms of improving the performance of sensors based on UOx, Bhushan et al. [104] proposed a new design from another perspective. This study constructed a bio-enzymatic biosensor for the detection of uric acid in wound fluid. The electrochemical electrode was based on a nanocomposite of multiwalled carbon nanotubes (MWCNTs) and Au nanoparticles (AuNPs), which was modified with UOx as a biocatalyst for uric acid oxidation and horseradish peroxidase (HRP) for electron transfer. The combined use of these two enzymes and the synergy between MWCNTs and AuNPs doubled the response. The biosensor exhibited the lowest detectable concentration of 9.91 μM with a sensitivity of 2.5 nA/μM.

Although these studies [62,97,98,99,100,103,104] have made some innovations in the design of the UOx sensor, they still lack consideration of the wearability and comfort of the sensor. Although there seems to be a contradiction between mechanical flexibility and electrical conductivity, a compromise between the two should be diligently explored, which will be one way to push the smart bandages into practical applications.

These studies adopted the screen-printing process in the production of electrodes, because this process is simple and cheap. However, the material commonly used for wound bandages is gauze, and the screen-printing process makes it difficult to print the electrode material on a loose substrate, as gauze has many pores and surface texture. In addition, the screen-printed electrode also has the risk of fatigue damage due to the mechanical strain caused by the frequent movements of the wearer. In response to this issue, Liu et al. [105] used a polyester thread soaked in carbon or Ag/AgCl ink as a raw material to embroider an electrochemical three-electrode system on gauze using an embroidery machine to construct a gauze-based embroidery sensor for detecting uric acid in wounds, which was also modified with UOx (Figure 2b). It had good specificity and a good linear relationship in the range of uric acid concentration of 0–800 μM. At the same time, it still maintained a high linear response (*R^2^* = 0.994) after folding and flattening 100 times, which reflected its good mechanical properties. Unlike the idea of Liu et al. [105], Sharifuzaman et al. [106] applied 2D MXene nanosheets to functionalize 3D laser-guided graphene (LGG) sheets via a C–O–Ti covalent crosslink (Figure 2c), and they transferred the obtained LGG-MXene hybrid scaffold onto polydimethylsiloxane (PDMS) to make a smart bandage for wound uric acid detection (Figure 2d). The crosslinking of the two materials improved the high sheet resistance and poor mechanical properties of the LGG chip. The sensor had a sensitive response to uric acid in the range of 50–1200 μM, and the sensitivity could reach 422.5 μA mM^−1^·cm^−2^. Moreover, the integrated bandage could also detect pH and temperature. Sharifuzaman’s study also enhanced the mechanical performance of the electrode while ensuring the electronic conductivity of the sensor, but another problem that is easily overlooked is that the biological macromolecules present in the wound may be close to the electrode surface and contaminate the electrode, thus reducing the service life and detection repeatability of the electrode [107].

This problem may be solved from two perspectives. On the one hand, a very low-cost disposable electrode can be designed to avoid this problem by replacing with a new electrode; on the other hand, some ingenious treatments can be made to the electrode, such that biological macromolecules cannot get close to the electrode surface, to avoid contamination of the electrode. Pal et al. [78] designed a low-cost disposable paper-based smart bandage from the first point of view to detect uric acid in wounds. They stencil-printed working and counter electrodes with carbon ink and a reference electrode with Ag/AgCl ink on Whatman #1 paper sprayed with a 2% solution of fluorinated alkyl trichlorosilane (RFSiCl_3_) in iso-propyl alcohol (IPA). The working electrode was modified with uricase. The sensor had a sensitive response to uric acid in the range of 0.22–0.75 mM, and the detection limit could reach 0.2 mM. It is worth mentioning that, although the low-cost paper-based electrodes were disposable, the micro wearable wireless potentiostat with the size of a coin developed by the team could be reused, which further reduced the manufacturing cost. However, from the perspective of patient compliance, reusable and regularly measurable wound smart bandages would be more acceptable to patients and doctors [26]. Jarošova et al. [72], from the second point of view, inkjet-printed a layer of carbon nanotubes (CNT) on Kapton substrate and coated it with nanoporous polyacrylamide (PA) hydrogel to detect the concentration of uric acid in the wound fluid. The hydrogel layer was rich in nanopores, which allowed small molecules to diffuse to the electrode surface for detection while preventing the access of larger “fouling” molecules, resulting in good selectivity, repeatability, and service life (Figure 2e). The linear range was 100–1000 μM/L (*R^2^* = 0.9997) with a sensitivity of 2.83 mA·L/mol.

##### The Detection of pH

pH is another key marker in wounds that is as important as uric acid. In smart bandages based on electrochemical detection, pH is usually not used as a separate target but in conjunction with other parameters to reflect the condition of the wound. Moreover, it can also be used as a calibration parameter for other electrodes [79]. Most researchers prefer potentiometer-type sensors to detect pH in wounds. Polyaniline (PANI) and its complex are usually used to modify the working electrode for pH detection, and Ag/AgCl is used to construct the reference electrode. PANI is a polymer that exists in three different oxidation states, one of which is known as the emeraldine form [108]. PANI will undergo protonation and deprotonation in acidic and alkaline environments, which will cause a change in potential difference between the two electrodes. Specifically, in the process of protonation, the combination of hydrogen ions and empty nitrogen sites will reduce the conductivity, thus reducing the open-circuit potential of PANI relative to the solution reference electrode. Therefore, PANI is suitable and widely used in pH detection [109]. For example, Sharifuzaman et al. [106] constructed a two-electrode system for pH detection, using cyclic voltammetry in aqueous hydrochloric acid to polymerize PANI on the working electrode based on LGG-MXene. The sensitivity of the sensor could reach −57.09 mV/pH at a pH value in the range of 4–9. Mostafalu et al. [110] prepared the PANI emeraldine-based membrane on the screen-printed carbon electrode, and then introduced H^+^ via HCl fumes into the PANI emeraldine-based membrane in a vacuum chamber to convert it into PANI emeraldine salt with high conductivity. The sensor used the potentiometric measurements to detect pH, which had good linearity in the pH range of 4–10 with a sensitivity of −50 mV/pH.

**Figure 3:**
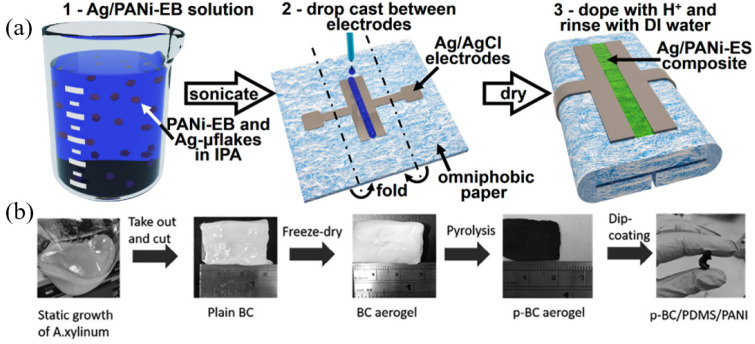
Modification of electrochemical sensors electrodes for pH detection in wounds. (**a**) Preparation process of Ag/PANI composite electrode [78]. Copyright (2018) Elsevier. (**b**) Fabrication process of p-BC/PDMS/PANI nanocomposite [112]. Copyright (2021) Elsevier.

The use of PANI is not limited to these traditional methods. Lyu et al. [111] successively coated the polyester thread with carbon ink and dipped it in PANI solution, before sewing the treated polyester thread onto the bandage to create a flexible sensor for wound PH detection. The PANI-functionalized thread was knotted using an overhand knot to create the structure of high surface area. The vertical 3D structure of this sensor made it more closely contact the uneven wound surface, speeding up the response time of the sensor. When the potentiometric measurements were used for detection, the sensitivity of the sensor could reach −40 mV/pH, and the response time was less than 40 s. Pal et al. [78] modified the pH-responsive silver micro flakes and PANI polymeric composite on the working electrode used for wound detection (Figure 3a). Polyaniline emeraldine salt gradually transforms into polyaniline emeraldine base with higher resistance under alkaline environment. Moreover, the silver microflakes reduce the resistivity of PANI, making it possible to measure the change in resistance at low voltage (100 mV) and, thus, accurately measure the PH in wound exudate. Therefore, the pH can be determined by detecting the impedance of the sensing electrode, with a linear detection range of 5.5–8.5. Yang et al. [112] prepared an innovative sensor based on a flexible p-BC/PDMS/PANI nanocomposite material for pH detection in chronic wounds (Figure 3b). The sensor used a 3D free-standing conductive carbon nanofiber aerogel derived from pyrolyzed bacterial cellulose (p-BC) as conductive substrate, which was combined with the proton-selective PDMS/PANI composite material. The sensor showed good stability, and the sensitivity was 50.4 mV/pH in the buffer with pH range of 4–10, while the sensitivity was 29 mV/pH in the simulated wound fluid with a pH range of 4–7.

By compounding other substances with PANI, the performance of traditional PANI-based electrochemical electrodes can be improved. However, other materials different from PANI have been reported for the modification of wound pH detection electrodes. For example, McLister et al. [113] modified poly-l-tryptophan on a substrate of carbon fiber electrodes wrapped in flexible polyester laminate sheath, and then studied the pH response of new redox wire prepared from the peptide homopolymer of tryptophan. The peak position of the electrogenerated quinone functional group generated by this material could be responsive to pH. The sensor produced a response in the range of pH 3–8 with a sensitivity of −59 mV/pH. In contrast, Mariani et al. [114] recently reported a new type of two-terminal sensor for pH detection based on chemically synthesized IrOx (a transition metal oxide) particles embedded in a semiconducting polymer poly(3,4ethylenedioxythiophene) (PEDOT) doped with poly(styrene sulfonate) (PSS) thin film. Due to the spontaneous redox reaction, the conductivity of this new type of composite material reversibly changed with the change in pH, enabling a more significant sensing performance in terms of repeatability, stability, and accuracy. The sensor showed a sensitivity of 59 ± 4 μA/pH in the range of pH 6–9.

##### The Detection of Skin and Wound Impedance

Compared to uric acid and pH, which reflect bacterial infection of the wound, skin or wound impedance has other valuable applications.

From an electrical point of view, a cell can be equated as an ion-rich center (intracellular fluid) separated by a relatively nonconductive barrier (cell membrane) in an ion-rich medium (extracellular fluid). The ability of these ion-rich media to conduct charge can be equivalently described in terms of electrical resistance; similarly, the barrier to charge flow (cell membrane) can be modeled in terms of capacitance, which is the equivalent of cell or tissue electrical impedance. Changes in biological structure can lead to changes in their equivalent impedance. For example, cell damage in tissues leads to rupture of the cell membrane and outflow of cytoplasm, allowing ions and currents to pass through the cell membrane. As a result, damaged cells will exhibit higher conductivity and smaller capacity to store charge, and they will exhibit a phase angle closer to zero in impedance measurements [115]. Impedance spectra are obtained by measuring impedance at multiple frequencies. EIS has a well-established measurement model; thus, it can be applied to wound detection, such as early warning of pressure sores or assessment of wound surface status. In practical measurements, the electrode surface material, electrode–skin contact area, electrolyte composition, and measurement frequency are the key factors for optimizing the impedance measurement system [116]. Swisher et al. [115] used inkjet printing technology to create electrode array patterns from gold nanoparticle ink on flexible polyethylene naphthalate substrates for early skin pressure ulcer detection, and they explored different models of pressure ulcer in rats. The sensor can be sensitive enough to detect minor, reversible changes in early skin pressure ulcers that are not obvious to the naked eye. It is worth noting that the relationship between pressure ulcers worthy of vigilance and their corresponding specific impedance thresholds has not been established. Although changes in skin pressure ulcers can be monitored, there is no reasonable parameter to report when to be vigilant, depending not only on new discoveries and advances in medicine, but also on differences in the detection systems and models used by each assay. Pal et al. [78] constructed a paper-based electrochemical sensor system to detect uric acid and pH in wounds, as described above. However, it should be emphasized here that the system also supported the measurement of skin impedance to warn of the appearance of early pressure ulcers. The sensor was verified using a rat pressure ulcer model, and it was found that the system can provide the greatest contrast between healthy tissue and damaged tissue in the frequency range of 40–60 kHz. The study also verified that damaged tissue will reduce impedance even more.

The application of EIS in skin and wound detection is not limited to pressure ulcer detection; the change in wound area can also be reflected by impedance detection [55,116,117,118], such that the healing of the wound can be known without frequently opening the wound bandage. Kekonen et al. [117] used a screen-printing process to prepare a flexible electrode array for wound healing monitoring on a polyurethane (PU) substrate, which contained 25 circular working electrodes and four reference electrodes. The detection system was used to detect the skin and wounds of subjects. The test results found that the measured impedance value caused by the wound dropped sharply; furthermore, with the shrinkage of the wound area and the formation of new epithelial tissue on the wound surface, the impedance value first recovered slowly and then increased rapidly. In a subsequent study [55], the team further optimized the system, compared the effects of multiple measurement frequencies, and performed continuous monitoring of the wound for a long time (142 h). It was found that skin impedance is easily affected by skin moisture level, skin thickness, and skin physiological state under the condition of low measurement frequency, while the stability of skin impedance over time is higher at the higher measurement frequency. Therefore, the measurement frequency should be weighed according to the actual detection system and target model. Kekonen et al. [116] recently constructed a 4 × 4 electrode array to study the changes in wound impedance with the development of wounds in patients with venous ulcers. The study carried out wound impedance testing on seven patients with venous ulcers and derived impedance data as the wound status index (WSI) to describe wound healing. According to the experimental results, there was a strong linear correlation between WSI and wound area. The wound state index established by this study has a reference value and promotes the process of quantifying the wound healing state. Pei et al. [118] used a magnetron sputtering process to prepare a star-shaped electrode array containing five electrodes for impedance detection of damaged skin, and the feasibility of the system was verified by in vitro experiments on pig skin and monitoring of chronic wound healing in live pigs. The result showed that the system had good impedance measurement accuracy and repeatability, and the wound could be easily distinguished by the bioimpedance of the wound.

##### The Detection of Other Biomarkers

In addition to uric acid, pH, and impedance, some biomolecules have been shown to be more directly related to wound state.

*P. aeruginosa* is a common strain in wound infection [14,119] and can be reflected by pyocyanin [120,121]. Pyocyanin has relatively active redox properties; thus, it can be detected by electrochemical methods. Sharp et al. [122] used carbon fiber as the base of the electrochemical working electrode to quantitatively detect pyocyanin in the wound simulation environment. The sensor had good linearity (*R^2^* = 0.998) in the range of pyocyanin concentration 1–100 μm. Since *P. aeruginosa* can survive in both aerobic and anaerobic environments, the study also verified that the sensor was not disturbed in any aerobic and anaerobic environment. Jarošová et al. [72] prepared polyacrylamide-coated carbon nanotube (PA/CNT) electrodes using an inkjet printing process and accurately measured uric acid and pyocyanin in a wound simulation solution at 37 °C. The use of PA can prevent other biological macromolecules from contacting the electrode, to avoid affecting the detection, as described above. The sensitivity of the sensor could reach 35.6 ± 0.8 mA·L/mol (*R^2^* = 0.9997) within the range of the concentration of pyocyanin 0.1–100 μm/L, and the detection limit was 0.10 μmol·L^−1^ (S/N = 3). In addition to the targeted detection of *P. aeruginosa*, the correspondence between pyocyanin and the reproduction status of *P. aeruginosa* can be used to explore the relationship between *P. aeruginosa* and related biochemical markers. Whether antibacterial and anti-inflammatory methods are effective on *P. aeruginosa* is another innovative application. Simoska et al. [79] constructed a carbon ultramicroelectrode array on a flexible substrate, which could detect uric acid, pyocyanin, and NO in the wound environment. The detection limit of pyocyanin was 1.0 ± 0.5 μM, and the linear dynamic range was 1–250 μM, while the detection limit of NO was 0.20 ± 0.05 μM, and the linear dynamic range was 1–100 μM. Subsequently, the study explored the corresponding relationship between pyocyanin and NO and the bactericidal effect of Ag^+^ on *P. aeruginosa*. The results showed that there was no direct corresponding relationship between pyocyanin and NO, and the addition of Ag^+^ would inhibit *P. aeruginosa*.

*S. aureus* is another common bacterial species in wound infections [123]. Most researchers expect to detect the uric acid content in the wound to reflect the reproduction of *S. aureus*; however, in fact, it is not only *S. aureus* that can cause a change in uric acid in wounds [124]. The inability to accurately determine the infection of the wound will result in the inability to predict the development trend of the wound more accurately, which may affect the treatment effect. Roy et al. [73] used a screen-printing process to print conductive carbon ink on flexible cellulose paper as a substrate and modified it with a composite of zeolitic imidazolate framework (ZIF 67) and carbon nitride (C_3_N_4_) conjugated with *S. aureus* probe DNA for detecting the DNA molecules of *S. aureus* in wounds, with a detection range of 1 Fm–10 μM. When DNA molecules of *S. aureus* bacteria hybridize with molecular probes on the electrode surface, the bulky double-helix structure formed has an obstructive effect on electron flow. The poor electron transport at the electrode–electrolyte interface leads to inefficient bilayer charging/discharging, which reduces the interfacial capacitance. The sensor adopted EIS, and the charge transfer resistance was directly proportional to the target DNA concentration of *S. aureus*. The limits of detection, sensitivity, and response time of the sensor at 21.04 kHz were 0.46 fM, 0.25 kΩ/fM/mm^2^, and 10 s, respectively. This undoubtedly provides an innovative method for the detection of *S. aureus*.

l-Tyrosine is another important biochemical marker of wounds. The sharp increase in the concentration of l-tyrosine in the wounds of diabetic patients can indicate the emergence of DFUs [72]. However, the current clinical routine detection methods for l-tyrosine, such as tandem mass spectrometry [125] and high-performance liquid chromatography (HPLC) [126], not only consume time and cost, but, more importantly, they also cannot achieve continuous in vivo detection, which will bring pain to patients. Fortunately, wearable electrochemical sensors for detecting l-tyrosine in wounds have recently been developed [127,128,129]. It is hoped that l-tyrosine can be detected in vivo without removing the bandage. Roy et al. [127] designed a new type of electrochemical sensor for l-tyrosine detection in the wound simulation environment. They used electrochemical anodization to prepare TiO_2_ nanotube matrix on the titanium foil, then constructed a reduced graphene oxide (rGO) film reduced by ultraviolet light irradiation (λ = 290 nm) to enhance the surface conductivity, and finally modified tyrosinase on the prepared TiNT—rGO hybrid electrode. They used EIS and electrochemical voltammetry to verify the performance of the sensor; the result showed that, when using EIS, the detection limit could reach 0.35 nM with a sensitivity of 0.106 Ω/nM/cm^2^, whereas, when using electrochemical voltammetry, the detection limit could reach 0.86 nM with a sensitivity of 1.986 mA/nM/cm^2^. Although the study achieved good performance in the detection of l-tyrosine, the sensor is not equipped with portable electronic equipment. In the follow-up study by Roy et al. [128], a wearable electrochemical sensor based on an α-MnO_2_/tyrosinase bio-enzyme for real-time monitoring of l-tyrosine was proposed and equipped with a portable electronic device containing a wireless communication module, which further pushed the sensing system into practical use. The detection limit and sensitivity of the sensor were 0.71 nM and 0.67 μA/nM/mm^2^ in the range of l-tyrosine concentration of 5 nM–500 μM with good linearity. Similar to Roy et al. [127], Bala et al. [129] reported an electrochemical enzymatic sensor for l-tyrosine detection based on a TiO_2_ nanotube film modified by a low-energy ion beam containing nitrogen ions and gold ions. Ions were deposited in the space between the vertically arranged self-organized titanium dioxide nanotubes, resulting in thickening of the tube wall, which enhanced the performance of the sensor, and the sensor could adapt to the wide detection range of 100 fM–500 μM. Although these wearable electrochemical sensors for l-tyrosine detection have been developed, they all rely on l-tyrosinase. Due to the instability of the enzyme, the promotion of this type of sensor to practical applications may be limited. Excellent non-enzyme-based wearable sensors for the detection of l-tyrosine need to be developed.

##### The Detection of Multiple Parameters

**Figure 4:**
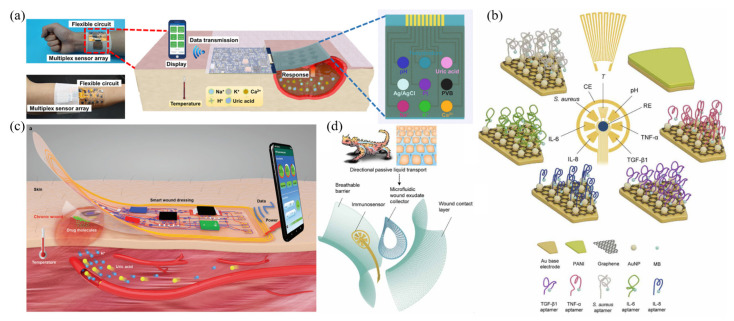
Flexible wearable sensors for wound multiparameter detection. (**a**) Schematic diagram of a wearable flexible sensor for detecting Na^+^, K^+^, Ca^2+^, pH, uric acid, and temperature in wounds [82]. Copyright (2021) American Chemical Society. (**b**) An integrated electrochemical sensor detect inflammatory mediators (including tumor necrosis factor-α, interleukin-6, interleukin-8, and transforming growth factor-β1), microbial burden (*S. aureus*), and physicochemical parameters (temperature and pH) [83]. Copyright (2021) Amer Assoc Advancement Science. (**c**) Smart dressing for pH, uric acid, and temperature detection in wounds and electronically controlled release of drug molecules for treatment [25]. Copyright (2021) John Wiley and Sons. (**d**) Sensor module for multiparameter wound detection, including a wound contact layer, microfluidic wound exudate collector based on the Texas lizard skin bionic principle, immunosensor, and breathable barrier [83]. Copyright (2021) Amer Assoc Advancement Science.

The wound environment is very complex, and it is often difficult to comprehensively evaluate the wound condition with a single parameter. Some researchers have recognized this problem and made efforts to address it. Mostly, this type of system has a high degree of integration; thus, the preparation of this type of sensor often requires micro-nano processing technology. Liu et al. [82] recently reported a flexible integrated multi-channel electrochemical sensing bandage for real-time monitoring of Na^+^, K^+^, Ca^2+^, pH, uric acid, and temperature at the wound site, providing a comprehensive and quantitative basis (Figure 4a). The preparation of the sensor relied on magnetron sputtering technology, with Au, Ag, and Pt as the electrode substrate, which had good electrical conductivity. The Na^+^, K^+^, and Ca^2+^ working electrodes were modified with ion-selective membranes that are sensitive to target ions. The response principle is based on the corresponding ion-selective membrane potential changes caused by changes in ion levels. The pH detection electrode was modified with pH-sensitive poly(3,4-ethylenedioxythiophene) polystyrene sulfonate (PEDOT:PSS), the uric acid electrode was constructed on the basis UOx, and Prussian blue was used as the catalytic medium in the intermediate step. The sensor had a sensitivity of 64.67 mV/pd in the range of Na^+^ concentration of 5–160 mM, a sensitivity of 61.59 mV/pd in the range of K^+^ concentration of 1–32 mM, a sensitivity of 29.26 mV/pd in the range of Ca^2+^ concentration of 0.01–100 mM, a sensitivity of 47.33 mV/pH in the range of pH 4–10, a sensitivity of 1.01 μA/mM in the range of uric acid concentration of 0–1000 μm, and a sensitivity of 0.16 Ω/°C in the range of temperature of 25–45 °C.

The biochemical markers in wounds that most researchers pay attention to are limited to uric acid, pH, and impedance (as mentioned above), whereas indicators of inflammatory mediators and bioburden also have important clinical value [130,131,132]. Gao et al. [83] constructed a wearable electrochemical sensor for multiple analyses of wound microenvironment, inflammation, and infection status (Figure 4b). The platform included a microfluidic wound exudate collector based on the bionic principle of Texas lizard skin (Figure 4d), an integrated electrochemical system that can quantitatively detect inflammatory mediators (including tumor necrosis factor-α, interleukin-6, interleukin-8, and transforming growth factor-β1), microbial burden (*S. aureus*), and physicochemical parameters (temperature and pH), and a portable circuit device. The system was used in a rat wound model to verify its detection performance and good biocompatibility, and the application on the wounds of five patients with active venous ulcers proved the feasibility of the design. In addition, a smart bandage closed-loop system that integrates detection and treatment functions has also been gradually developed. For example, Xu et al. [25] recently proposed an integrated and wearable flexible intelligent bandage that can not only detect parameters such as pH, uric acid, and temperature in the wound, but also electronically release drugs to inhibit bacterial reproduction (Figure 4c). It is worth mentioning that they constructed a carbon-based working electrode and counter electrode, as well as a reference electrode based on Ag/AgCl, and they used a nonenzymatic way to detect uric acid. Specifically, they electrodeposited a composite of rGO and AuNPs on the working electrode by cyclic voltammetry and used differential pulse voltammetry to detect uric acid. The results showed that this design could sensitively distinguish the redox peak of uric acid and other interferences (such as ascorbic acid), and it also avoided the problem of poor sensor stability caused by the use of UOx. The sensitivity and detection limits of the sensor for uric acid were 0.04428 μA/μM and 3.11 μM, respectively, and the sensitivity and detection range for pH were 60.34 mV/pd and 3–10.

Table 1 summarizes the detection methods, sensor materials, fabrication processes, and features of the flexible wearable sensors for wound detection with electrochemical detection principles mentioned in this paper.

#### 3.1.2. Other Electrical Detection

Compared with electrochemical methods, there are also some methods based on fundamental electrical theory. Utilizing the electrical properties of the micro or macro structure of special materials or the inherent electrical characteristics of the wound, accurate wound conditions and the phase judging of wound recovery can be quantified in an integrated system.

**Figure 5:**
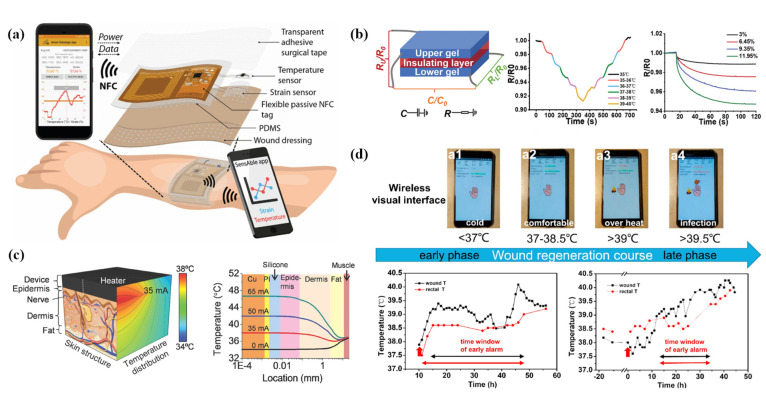
(**a**) Overview of the NFC-based smart bandage for wireless strain and temperature real-time monitoring [134]. Copyright (2020) IEEE. (**b**) Schematic illustration of the sandwich-structured sensor. The resistance curves with temperature change from 35 to 40 °C and back to 35 °C, and those with compressive strain change from 3% to 11.95% [135]. Copyright (2021) John Wiley and Sons. (**c**) Results of modeling the bioheat transfer equation and the calculated temperature distribution for different input currents [136]. Copyright (2014) John Wiley and Sons. (**d**) Visual indicator and alarm interface of the customized app, which would report visual wound status of corresponding wound temperature. The time–temperature curve of early-phase and late-phase infection model and rectal temperature was monitored as a control (red curve) [137]. Copyright (2020) Elsevier.

A large number of trauma real-time monitoring studies concentrated on the conductive composite polymer materials, which exhibit excellent impedance characteristics to temperature, humidity, and strain. Additionally, current developments of printed electronics have reduced the process and time costs, prompting more potentially sensitive materials to be further encapsulated into miniature flexible sensing units. Honda et al. [86] printed a PEDOT:PSS/CNT hybrid material temperature sensor using a PDMS mask, with a sensitivity of 0.61%/°C (RT ~ 50 °C). The result was better than these two single materials, which may be owed to the electron hopping at the interface between these two substances. However, due to the process cost of printing technology, the size of the sensing unit is large and not friendly for daily wear needs. Descent et al. [133] spin-coated temperature- and humidity-sensitive graphene oxide on the interdigitated electrodes printed using thermal transfer and donor ribbon technology. The temperature and humidity sensors were well fitted by a linear (*R^2^* > 0.99) and quadratic polynomial (*R^2^* ≈ 0.99) relationship. Escobedo et al. [134] similarly selected PEDOT:PSS as both a temperature- and a strain-sensing material (Figure 5a). The temperature sensitivity reached about 1.2%/°C (25–90 °C). As for the strain, the trauma–normal detection range was 150% per free curvature and 12 k% per stretch percentage. A novelty multi-responsive hydrogel was created by combining temperature-sensitive *N*-isopropyl acrylamide (NIPAAm) and glucose-sensitive methylacrylamide phenyl boric acid (MPBA) on zwitterionic carboxyl betaine [135]. The sensing section could be regarded as two resistance sensors and one capacitance sensor, simultaneously responding to temperature, glucose, and strain (Figure 5b). The final system had a good quadratic fitting relationship with temperature (*R^2^* > 0.99) and exhibited a significant resistance change to the blood glucose concentration of 0.5% to 5%, including the range in the wounds of diabetic patients. This research demonstrates the possibility that the materials, which are sensitive to molecules related to wound state, can be integrated into the sensor system.

Not limited to impedance characteristics, in the field of monitoring traumatic strain, other features are also applied in electrical detection scenarios. Hosseini et al. [138] first tried to use biodegradable piezoelectric glycine to fabricate flexible electronics, embedding piezoelectric γ-glycine microcrystals into PDMS. External pressure could drive the sensor to the output voltage and generate a small amount of power. Despite its inadequate work integrity, the proposed voltage therapy and piezoelectric energy storage ideas are worthy of in-depth implementation. Moreover, toward pressure sensing based on capacitor change, the studies of medium layer transmitters and structures to improve the dielectric constant or its variation gradient incorporate micro capacitor sensors for wound detection. Wang et al. [139] focused on the combined effect of silver nanowire (AgNW) and PU in improving the dielectric constant between capacitor plates. High concentrations of AgNW doping could greatly increase the sensitivity of system capacitive sensing to 5.54 kPa^−1^. Because of its size and flexibility, it could fit in various positions on the human skin and could detect multiple scenes such as bending of fingers or knees and muscle movements. Deng et al. [140] processed strain-responsive and -sensitive PDMS into an array pyramidal structure as the capacitive medium. Simultaneous variation of the distance between the plates and the relative dielectric constant promoted its sensitive response. In the range of 0–200 mmHg, the sensitivity was 270.8 kHz/mmHg. Parasitic capacitance introduced by skin in practical applications was considered and the calibration was completed on fresh pigskin. When measuring the capacitance of a capacitive sensor, the wireless passive integrated LC resonant circuit becomes the first choice with external receiving coils for convenient acquisition of exact pressure changes. Preliminary exploration on the LC resonant circuit was conducted [86], but experiments only tested the difference of the circuit resonant frequency of the interdigitated capacitor with or without finger touch. Farooq et al. [141] established comparative experiments on different size coils, test ranges, and other factors. Detailed and quantitative analysis was performed on the selection of coil models and sensing variates.

While the above applications involve sensitive materials in electrical monitoring, initiatory analyses have found that the dielectric properties of wound tissues are relevant to the trabecular evolution process. Charkhabi et al. [142] used the wound region as a self-capacitance medium to reflect the state of the wound with the help of a passive LC resonant circuit. During the healing process, the monitoring parameters such as the amplitude of the scattering parameter S_21_ and the resonance frequency gradually rose. However, due to the small number of experimental samples, as well as the influence of coil process errors and signal noise, no precise calibration curve was obtained. Zhang et al. [143] worked up a simplified wound model to build a sensor with the coplanar waveguide principle. In the model, the reflection impedance matrix was found to contain information about the dielectric properties of the measured tissue. However, the whole analysis is set on a simulation and requires more actual measurements.

The majority of the mentioned studies lacked ample experimental exploration before practical applications. In fact, commercial or semi-finished sensors are also frequently integrated into electronic systems for monitoring the status of wounds and applied in animal or human wound sensing experiments. These articles focused on obtaining the correspondence between the wound evolution process and the monitoring variates. They expect to build accurate models which contribute to clinical research, evaluation, and various intervention methods. In this case, the digital electrical signal of the sensor is continuously recorded and the potential relationship between the selected typical data with the number of days as a variable and the corresponding camera imaging sample or other reliable detection results are contrastively analyzed. Hattori et al. [136] proposed a skin-like electronic system with a soft mechanical texture of the epidermis, with a fractal layout of thermistor and heating devices. Experiments tested the human body postoperative incision slice, established the skin near the wound at normal skin temperature, along with thermal conductivity of the time and space mapping (Figure 5c). Similarly, Zhang et al. [144] designed a three-layer smart dressing, including a biomimetic nanofiber membrane, an electronic system containing temperature and humidity sensors, and an ultraviolet crosslinked hydrogel. The temperature change curve of the full-thickness wound on both sides of the back of New Zealand rabbits was observed. It was found that the local temperature was slightly higher for 3–4 days before the wound, which may represent the hyperemia and inflammation stage of wound healing. As the wound entered the repair stage, the local temperature dropped and reached a stable plateau. Then, more detailed studies [137,145] were carried out: a double-layered band-aid detection and drug delivery system were designed. The upper and lower layer contained the STH21 temperature sensor and the healing medicine to be released, respectively. Experiments were on pigs (skin structure and healing mechanism are very similar to humans) with full-thickness incision wounds on the back surface. United with the regeneration, wound temperature fluctuation was attributed to three main phases (rising stage: below 39 °C, platform infection stage: 39–39.5 °C, and falling stage: below 39 °C). In the wound histological examination, it was found in a timeline that the unhealed area was dominant, and then the ratio of the two was close; eventually, the healed area was dominant. Furthermore, it was found that the number of inflammatory cells with changed appearance and the number of newly formed blood vessels were also similar to the trend of wound temperature fluctuations. The corresponding intelligent early warning system and active drug delivery feedback were developed according to the law (Figure 5d).

Many studies established a multi-sensor integrated detection platform, whose sensitivities reached the advanced standard after optimization. Nonetheless, due to the lack of practical experiments, the negative effects of practical applications cannot be encountered. Furthermore, there is still no combined analysis of multiple state variates in these actual experiments of multi-sensing, such that the significance of the multi-sensing system cannot be reflected. Ultimately, multi-sensor systems will inevitably encounter hardware circuit size and performance tradeoffs in the procedure of micro-integration. The challenge of merging multi-sensor interfaces may also require a technical break to innovate. From the perspective of hardware signal reading, Cho et al. [146] fabricated an integrated chip, enabling the monitoring of acquisition voltage, current, and capacitor sensor data in time-sharing mode.

Table 2 summarizes the detection principles, sensitive materials, and features of the flexible wearable sensors for wound detection with other electrical detection principles mentioned in this paper.

### 3.2. Optical Detection of Wound Markers

Optical inspection methods have the advantage of simplicity and low cost compared to electrical inspection methods, and the physiological information in the wound can be converted into an optical signal for direct observation. Meanwhile, some smart bandages based on optical principles can use mobile phones for data acquisition, combined with image processing algorithms to complete the analysis of the optical signal, which allows optical inspection to be done more accurately for wound assessment.

Saminathan et al. [147] developed an efficient algorithm using asymmetry analysis and computer-assisted diagnostic system to detect the diabetic foot with infrared thermal images. The results showed the support vector machine classifier has an accuracy of 95.61%, sensitivity of 96.5%, and specificity of 92.41%. However, this study did not incorporate wearable devices. Gamerith et al. [148] presented a pH-responsive indicator that changes from yellow to green to blue with rising pH. They demonstrated sensing swabs that could simultaneously be used for wound cleansing and pH sensing, thereby providing important information about the wound status. This pH sensor allowed the detection of increased infection risk in sampled wound fluids. Quick and preliminary judgment can be made using this approach in terms of the infection of the wound. It has the advantages of low cost and convenience, but it cannot provide accurate and quantitative analysis. Yang et al. [149] synthesized novel orange-emissive carbon quantum dots (O-CDs) and combined them with cotton cloth as a wound bandage. The usage of the O-CD-coated bandage to detect pH is free from the interference of blood contamination and long-term storage. Accordingly, wound pH can be both predicted theoretically and estimated visually in the case of blood contamination and long-term observations. However, this method is still unable to perform quantitative analysis of in vivo wound infection in real time. Panzarasa et al. [150] proposed a semiquantitative detection method of wound pH (i.e., 5.5–7.5) via colorimetric and fluorescence intensity calibration of the synthesis of a new ion pair between pyranine and benzalkonium. Using a smartphone camera as a spectrometer, semiquantitative results of pH could be obtained. This provides users with a tool to initially distinguish the wound status without standard testing equipment, which would be very important, especially for home medical or community medical care.

**Figure 6:**
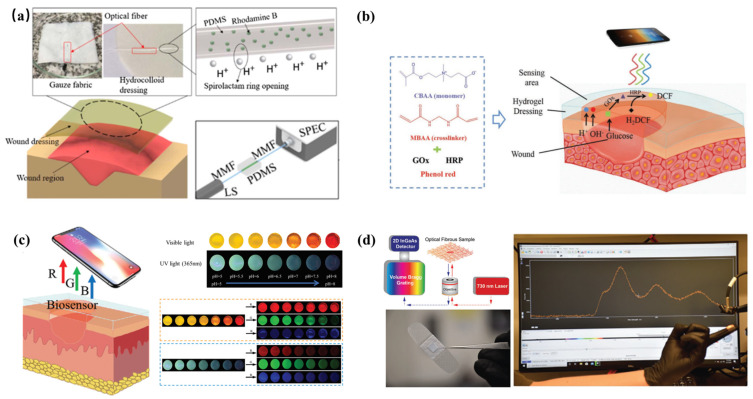
Portable wound detection devices using optical detection methods. (**a**) Schematic of quantitative detection of pH and pressure using the spectrometer USB2000+ [151]. Copyright (2021) Chinese Laser Press. (**b**) A photographic method to establish functional relations between glucose/pH and RGB intensity using a smartphone [152]. Copyright (2019) John Wiley and Sons. (**c**) A remote approach of dynamic wound status evaluation with the RGB analysis of the images collected by smartphones [154]. Copyright (2021) John Wiley and Sons. (**d**) A wearable wound bandage attached the microfibrous platform for in situ monitoring of peroxide [157]. Copyright (2021) John Wiley and Sons.

In the study of quantitative analysis, Junior et al. [151] presented the development of the first optical fiber-embedded smart wound dressing using a PDMS precursor doped with rhodamine B dye for pH assessment (Figure 6a). Meanwhile, the smart bandage also enabled the assessment of pressure on the wound region due to the low Young’s modulus of PDMS. The optical signals were transmitted by the optical fibers and acquired by the spectrometer USB2000+ (Ocean Optics, Douglas Avenue Dunedin, FL, USA) to realize the quantitative detection of pH 4–7 and pressure 0–0.3 kPa. This method achieves quantitative analysis via spectrometer, but it relies on optical fibers and the host computer software and, therefore, lacks portability and compactness.

To achieve portability of wound detection, Zhu et al. [152] developed a multifunctional zwitterionic hydrogel dressing to detect the pH range of 4–8 and glucose level of 0.1–10 mM simultaneously for diabetic wound status monitoring (Figure 6b). A pH indicator dye (phenol red) and two glucose-sensing enzymes (glucose oxidase and HRP) were encapsulated in the hydrogel dressing. They also developed a photographic method to establish functional relations between glucose/pH and RGB intensity and the color and fluorescence signals could be quantified by the smartphone for precise measurement without the assistance of large equipment, making it possible for this multifunctional wound dressing to be used in the community to better guide the wound care. In another study using a smartphone for wound analysis, García et al. [153] proposed a colorimetric sensory polymer film that changes its color upon contacting amino acids, which allows for the quantification of the overall amino-acid concentration in a pH range of 3–11 by simply analyzing the photograph taken with a smartphone. The digital pictures were analyzed with a generic image software to obtain the RGB of the entire surface of the sensory disc and calculate the amino-acid concentration by only taking a photo. Untrained personnel could operate to obtain the quantification of amino acids because of the simple experimental procedure. Moreover, the sensor could provide an analytical tool not subjected to subjective evaluation to help diagnose human chronic wounds without complex reactants or expensive equipment. The use of smartphones can not only achieve in vivo monitoring but also perform real-time remote evaluations. Zheng et al. [154] presented multifunctional double colorimetry-integrated polyacrylamide–quaternary ammonium chitosan–carbon quantum dots (CQDs)–phenol red hydrogels to detect the wound pH level (Figure 6c). In the context of both ultraviolet and visible light, on-site wound pH signals could be accurately indicated through the RGB analysis of the images collected by smartphones; thus, dynamic wound status could be reflected for a real-time evaluation in a remote approach. This work revolutionized wound care in hospitals, primary care facilities, and community and family medicine. Wang et al. [155] proposed an inexpensive, reliable, and accurate tool for wound measurement combining a smartphone and corresponding app program. The application can record and track wound healing over time by identifying the edge of the wound, calculating the area of the wound, and detecting the skin temperature. The detection effect is equivalent to the clinically recognized reference thermometer. This work is of great significance for home monitoring, community testing, and telemedicine. Carrière et al. [156] presented a small, low-priced, and handheld thermal imager with excellent reliability. Through temperature measurement and comparison calculation of burn skin, accurate assessment and judgment of burn wound healing can be achieved using this handheld thermal imager. The handheld feature makes it possible for the patient or the diagnosing doctor to carry the imager and monitor at any time.

Although these studies made some breakthroughs in terms of portability, they are still lacking in the realization of wearable wound monitoring equipment. Safaee et al. [157] made important innovations in the portability and wearability of wound monitoring sensors. They utilized a one-step coaxial electrospinning process to fabricate microfibrous textiles incorporating peroxide-sensing single-walled carbon nanotubes and attached the microfibrous platform onto a commercial wound bandage for in situ monitoring of peroxide (Figure 6d). Furthermore, the wearable optical platform was able to detect peroxide wirelessly and reversibly in a physiologically relevant range for wounds (1–250 μm), without any electronics or power sources embedded. Although, at this stage, they utilized a benchtop near-infrared probe spectrometer for signal acquisition from the samples, they aim to miniaturize the external unit by assembling a thermoelectrically cooled InGaAs photodiode, an LED light source, and bandpass filters to create portable and wearable versions of the detection platform in future work. This wearable bandage is essential to enable not only point-of-care wound diagnostics for real-time monitoring in wound sites but also deliver accurate amounts of antioxidants and antibiotics to the wounds. Zhang et al. [158] also proposed a series of reversible thermochromic fibers (VTFs) with high portability. The VTFs were prepared via melt spinning, and VTF samples can reflect blue, red, and purple. Furthermore, these VTFs can be decolorized with the increase in temperature, before restoration to the original color within 60 thermal cycles. This provides great convenience for directly judging the change in skin temperature by observing the change in the color of the optical fiber, and a combination with other sensors will have greater potential.

Table 3 summarizes the detection methods, sensor materials, fabrication method, and features of the flexible wearable sensors for wound detection with the optical detection principles mentioned in this paper.

## 4. Conclusions and Outlook

In the paper, we reviewed the specific application of optical and electrical methods in wound marker detection. It is easy to see that there is an ongoing investigation into methods for the detection of key wound markers such as uric acid, pH, and temperature. Researchers have explored improved methods of detection schemes from a variety of perspectives and have achieved increasingly better results. Considering that the application scenario of smart bandages is fragile wounds, some researchers have actively explored substrate materials with good biocompatibility, softness, and comfort, and they have tried various methods to improve the mechanical properties and electrical conductivity of the sensors to obtain more accurate detection results. These efforts are commendable. Some recent studies have added the function of drug release on the basis of ensuring the detection ability, which makes the smart bandage form a complete closed loop for wound detection and treatment, further pushing the smart bandage to practical use.

However, we also need to face the problems that still exist. First of all, there are inadequate markers for wound detection, the corresponding relationship between the marker and the wound condition is not sufficiently clear, and the relationship between the markers is not explicit enough. This indicates that the basic research of wound physiology needs further exploration and discovery, and researchers need to conduct more experiments to establish the abovementioned relationship. Secondly, although some enzyme-based sensors can achieve higher specificity and sensitivity, the activity of enzymes is easily affected by other factors, resulting in smart bandages composed of these sensors potentially being unsatisfactory in terms of stability and repeatability. With the advancement of basic science, more materials for modification are being developed, which makes it possible to use new materials, such as nanomaterials, to replace enzymes. Lastly, we found that many studies still stopped at spiked detection in the wound simulation fluid instead of using the sensor in the biological model for further exploration. As a result, these researchers were unable to encounter the problems that may exist in the actual application of smart bandages and did not further think about how to solve these problems; therefore, these smart bandages cannot go further in the practical process. We believe that truly excellent smart bandages should be applied to actual biological models to verify their performance.

The current exploration of smart bandages is still in its infancy, but the idea of combining drug release with wound marker detection may drive smart bandage technology toward maturity. This not only reduces the patient′s pain and prevents the wound from deteriorating, but also saves a lot of medical resources and treatment costs, which we believe will certainly be one of the future trends in the development of smart bandages. When it comes to practicality, energy supply and signal transmission cannot be avoided, as they are crucial to the wider acceptance of smart bandages. There have been attempts to combine near-field communication (NFC) technology with smart bandages to give them the advantage of being passive wireless, allowing a further step forward in terms of practicality. We also believe that advanced physicochemical technologies should be actively combined with smart bandages. Specifically, due to the presence of multiple biochemical substances in the wound, biofuel cell technology could perhaps enable smart bandages to function without the need for external energy. In addition, the application of the Internet of things and 5G technology will also realize true “smart bandages”. Lastly, the ideal scenario for smart bandages is to realize telemedicine. With the help of smart bandages, patients with chronic wounds can enjoy a more comfortable and free life without frequent visits to the medical center or staying in the ward, relying on the combination of new technologies.

## Figures and Tables

**Table 1:** The detection methods, sensor materials, fabrication processes, and features of the flexible wearable sensors for wound detection with electrochemical detection principles mentioned in this paper.

Wound Marker	Detection Method	Sensor Material *	Fabrication Method	Feature	Ref.
Uric acid	SWV	Insulating laminate/carbon fiber mesh/cellulose acetate	Laser etching	Easy integration and stabilization	[97]
Uric acid	CV	Insulator/Prussian blue carbon ink/UOx	Screen printing	Wireless communication, applicable to mechanical deformation, good selectivity	[62]
Uric acid	CV	Commercial screen-printed carbon electrode/UOx entrapped in PVA-SbQ, FCA	-	Good selectivity and stability, (maintain 90% activity until the 5th day)	[103]
Uric acid	CV	Commercial screen-printed carbon electrode/nanocomposite of MWCNTs and AuNPs/UOx, HRP	-	High sensitivity and low detection limit	[104]
Uric acid	CV	Gauze/polyester thread soaked in carbon ink/UOx	Embroidery fabrication process	Wearing comfort, soft, good flexibility, and applicable to mechanical deformation	[105]
(a) Uric acid, (b) PH, (c) temperature	(a) DPV, (b) potentiometric measurement, (c) thermistor measurement	PDMS/LGG-Mxene/(a) UOx, BSA, (b) PANI	Laser scribing	Multi-marker detection, in-situ detection, smart stretchable, and flexible multifunctional	[106]
(a) Uric acid, (b) pH, (c) impedance	(a) Chronocoulometry, (b) impedance measurement, (c) EIS	Whatman #1 paper/(a) carbon/UOx, (b) carbon/silver microflakes and PANI polymeric composite, (c) Ag/AgCl ink/conductive hydrogel	Stencil printing	Low cost, flexible, breathable, multi-marker detection, detachable, and disposable	[78]
Uric acid, pyocyanin	SWV	Kapton substrate/carbon nanotube/nanoporous PA hydrogel	Inkjet printing	Good selectivity, repeatability, and service life	[72]
pH	Potentiometric measurement	Patch substrate/carbon/PANI	Laser-machining and screen-printing	Support drug release, Bluetooth communication	[110]
pH	Potentiometric measurement	Polyester threads/carbon/PANI	Stitching process	Low cost, biocompatible, soft, perception of deep and uneven wounds	[111]
pH	Potentiometric measurement	Nanocomposites of p-BC, PDMS and PANI	Pyrolysis aerogel	Lost cost, soft, easy fabrication, and mechanical robust 3D carbon nano-network structures	[112]
pH	SWV	Polyester laminate/carbon fiber/poly-l-tryptophan	-	Biocompatible	[113]
pH	Voltammetry	Conducting ink/chemically synthesized IrOx particles embedded in a PEDOT:PSS thin film	Screen printing	Good reproducibility, stability, and accuracy	[114]
Impedance	EIS	Polyethylene naphthalate substrate/gold nanoparticle ink/hydrogel	Inkjet printing	Flexible, stretchable, mechanical robustness, and in vivo detection of rat models	[115]
Impedance	EIS	Thermoplastic polyurethane substrate/silver ink/biomedical grade carbon ink	Screen printing	Long-term monitoring, in vivo detection of human	[55,117]
Impedance	EIS	PET/biomedical-grade carbon ink	Screen printing	Clinical applications, long-term-monitoring and sensitive	[116]
Impedance	EIS	PI substrate/Cu film/PDMS	Magnetron sputtering	Accurate, reliable, wireless communication, and in vivo detection of pig models	[118]
Pyocyanin	SWV	Insulating polyester sheath/carbon fiber	Laser-etched	Application to aerobic and anaerobic environments	[122]
Uric acid, pyocyanin, NO	SWV	Poly(ethylene terephthalate) (PET) substrate/layers of pyrolyzed photoresist film	Electron beam deposition, atomic layer deposition	Flexible, good selectivity, and multi-marker detection	[79]
the DNA molecules of *S. aureus*	EIS	Cellulose paper/carbon ink/composite of zeolitic imidazolate framework (ZIF 67) and carbon nitride (C_3_N_4_) conjugated with *Staphylococcus aureus* probe DNA	Screen printing	Cost-effective, disposable, portable, and specific	[73]
l-Tyrosine	CV, EIS	TINT-rGO/tyrosinase	Electrodeposition	High conductivity, robustness, biocompatibility	[127]
l-Tyrosine	CV	Commercial band-aids/carbon conductive ink/α-MnO_2_/tyrosinase bio-enzyme	Screen-printed	Good selectivity, wireless communication, stable	[128]
l-Tyrosine	EIS	TINT film/low-energy ion beam containing nitrogen ions and gold ions	Ion beam technique	Sensitive and wide detection range	[129]
(a) Na^+^, K^+^, Ca^+^, (b) pH, (c) uric acid, and (d) temperature	(a), (b) Potentiometric measurement, (c) chronoamperometry, (d) thermistor measurement	PET/Au/(a) PEDOT:PSS/ion-selective membrane,(b) PEDOT:PSS/polyaniline emeraldine (c) chitosan-Prussian blue mediator layer/UOx, (d) graphene	Magnetron sputtering	Multi-marker detection, sensitive, wireless communication, in vivo detection of rat models	[82]
(a) Tumor necrosis factor-α, interleukin-6, interleukin-8, transforming growth fac-tor-β1, *S. aureus*, (b) pH, (c) temperature	(a) SWV, (b) potentiometric measurement, (c) thermistor measurement	PU film/AuNPs-GP/(a) aptamer, (b) PANI/medical-grade PU film	Sputtering and photolithography	Multi-marker detection, biocompatibility, collection of wound exudates, wireless communication, in vivo detection of rat models	[83]
(a) pH, (b) uric acid, (c) temperature	(a) potentiometric measurement, (b) DPV, (c) thermistor measurement	PI substrate/carbon/(a) AuNPs/PANI, (b) rGO/AuNPs	Screen printing and laser cutting	Multi-marker detection, stabilized, supports drug release, wireless communication, in vivo detection of rat models	[25]

The meanings of the abbreviations in Table 1 can be obtained in Abbreviation table. * In the sensor material column, the order from left to right means that the sensor material is ordered from the bottom to top.

**Table 2:** The detection principles, sensitive materials, and features of the flexible wearable sensors for wound detection with other electrical detection principles mentioned in this paper.

Parameter	Detection Principle	Sensitive Material	Feature	Ref.
Temperature and pressure	Resistive and capacitive sensing	PEDOT:PSS/CNT hybrid material	Pioneer in the realization of printed sensors	[86]
Temperature and humidity	Resistive sensing	Graphene oxide	Printed interdigitated electrodes using thermal transfer technic	[133]
Temperature and strain	Resistive and capacitive sensing	PEDOT:PSS	High strain resolution	[134]
Temperature, strain, and glucose	Resistive and capacitive sensing	Zwitterionic thermo glucose-sensitive skin-like hydrogel	Continuous real-time monitoring of three indicators infection, swelling, and blood glucose	[135]
Strain	Piezoelectric effect	Piezoelectric γ-glycine micro-crystals	Biodegradable, potential for self-powered and autonomous electrical stimulation	[138]
Strain	Capacitive sensing	AgNW and PU	Flexible, suitable for different parts of the body	[139]
Strain	Capacitive sensing	Pyramidal PDMS elastomers	Fabricated using a silicon anisotropic etching mold	[140]
Dielectric properties of wound tissues	Capacitive sensing	Wound tissues	Detection of wound skin as self-capacitance medium	[142]
Dielectric properties of wound tissues	Coplanar waveguide	Wound tissues	A novel approach to wound assessment by transmission line theory	[143]

The meanings of the abbreviations in Table 2 can be obtained in Abbreviation table.

**Table 3:** The detection methods, sensor materials, fabrication methods, and features of the flexible wearable sensors for wound detection with the optical detection principle mentioned in this paper.

Wound Marker	Detection Method	Sensor Material	Fabrication Method	Feature	Ref.
Temperature	Thermal imaging	FLIR E60 thermal imaging camera	-	High accuracy and sensitivity	[147]
pH	Color indicator	Swabs or dressings	A silane-based coupling agent for immobilization of bromocresol purple	Low cost and convenience	[148]
pH	Colorimetric	Cotton cloth	Microwave-assisted heating of 1,2,4-triaminobenzene and urea aqueous solution	Biocompatibility, drug compatibility, resistance leachability, and high reversibility	[149]
pH	Fluorescence	Membranes and commercial wound dressings	Pyranine was incorporated in wound dressing via benzalkonium	Portable and semi-quantitative	[150]
pH and pressure	Spectroscopy	Spectrometer USB2000+	An intrinsically pH-sensitive optical fiber was fabricated using a polydimethylsiloxane precursor doped with rhodamine B dye	Portable and quantitative	[151]
pH and glucose	Colorimetry	Multifunctional zwitterionic hydrogel	Phenol red, GOx, and HRP were encapsulated in the polycarboxybetaine hydrogel matrix	Portable and quantitative	[152]
Amino acid	Colorimetry	Colorimetric sensory polymer film	Test kit with colorimetric sensory polymer film	Portable and quantitative	[153]
pH	Colorimetry	Hydrogels	Double colorimetry-integrated polyacrylamide–quaternary ammonium chitosan–carbon quantum dots (CQDs)–phenol red hydrogels	Portable, quantitative, real-time, and remote	[154]
Temperature	Infrared method	FLIR™ infrared camera	Combined with smartphone and the Swift Wound app	Inexpensive, easy to use, Reliable, and accurate	[155]
Temperature	Infrared method	Infrared thermography	Containing a lepton thermal sensor and a visible VGA imager	Small, low-priced, and handheld	[156]
Reactive oxygen species	Fluorescence	SWCNT nanosensors	Optical core–shell microfibrous textiles incorporating single-walled carbon nanotubes (SWCNTs)	Portable, wearable, and real-time	[157]
Temperature	Colorimetry	Reversible thermochromic fibers	Thermochromic microcapsules and polypropylene (PP) was used as color indicator and polymer fiber matrix	Portable and reusable	[158]

## Data Availability

Not applicable.

## Abbreviations

DFU	diabetic foot ulcer
ATP	adenosine triphosphate
*S. aureus*	*Staphylococcus aureus*
*P. aeruginosa*	*Pseudomonas aeruginosa*
UOx	uricase
EIS	electrochemical impedance spectroscopy
PB	Prussian blue
FCA	ferrocene carboxylic acid
MWCNTs	multiwalled carbon nanotubes
AuNPs	Au nanoparticles
HRP	horseradish peroxidase
LGG	laser-guided graphene
PDMS	polydimethylsiloxane
IPA	iso-propyl alcohol
CNT	carbon nanotube
PA	polyacrylamide
PANI	polyaniline
p-BC	pyrolyzed bacterial cellulose
PEDOT	poly(3,4ethylenedioxythiophene)
PSS	poly (styrene sulfonate)
WSI	wound status index
PA/CNT	polyacrylamide-coated carbon nanotube
HPLC	high-performance liquid chromatography
rGO	reduced graphene oxide
PEDOT:PSS	poly (3,4-ethylenedioxythiophene) polystyrene sulfonate
PU	polyurethane
NIPAAm	*N*-isopropyl acrylamide
MPBA	methylacrylamide phenyl boric acid
BSA	bovine serum albumin
PET	polyethylene terephthalate
PI	polyimide
TINT	TiO2 nanotube
AgNW	silver nanowire
O-CDs	orange-emissive carbon quantum dots
CQDs	carbon quantum dots
NFC	near-field communication
VTFs	reversible thermochromic fibers
